# Capgras syndrome in an elderly patient with severe organic comorbidities and aggression: a case report

**DOI:** 10.1192/j.eurpsy.2025.1769

**Published:** 2025-08-26

**Authors:** N. Zigic, N. Aljukic

**Affiliations:** Department of Psychiatry, University Clinical Center, Tuzla, Bosnia and Herzegovina

## Abstract

**Introduction:**

Caprags syndrome is a rare syndrome characterized by a false belief that an identical duplicate has replaced someone significant to the patient. It is widely regarded as the most prevalent of the delusional misidentification syndromes and appears in psychiatric and non-psychiatric cases, including organic disorders.

**Objectives:**

To present a case report of 84 years old male patient with severe organic comorbidities who developed Capgras syndrome.

**Methods:**

Psychiatric interview

**Results:**

An 84-year-old male patient came to the first psychiatric examination accompanied by his son, due to the suspicion and hostility he has been showing towards his wife for the past month. A few days before the examination, patient became extremely aggressive in the evening hours, he accused his wife that she was not his wife, that another person had been framed instead of her, he demanded that she show him her identity card and threatened to report her to the police. The wife locked herself in the bathroom in fear, but the patient broke down the door. Neighbors called the police, who then restrained him. The patient calmed down after that, but wife went to live with her son. During the examination, patient was completely calm and cooperative, with a neat appearance, oriented, his thought process was normal, conversation was conducted adequately in the desired direction. When asked about thought content he dissimulated it by stating that he was angry because his wife often hangs out with other women and doesn’t pay enough attention to him. He denied the presence of hallucinations. Affect was stable, cognitive capacities seemed appropriate for his age. Patient has been treated for several organic comorbidities, including prostate cancer, which was removed a few years ago, but due to problems with urination after surgery he wears a permanent catheter. He was diagnosed with atrial fibrillation and diabetes. He had a heart attcak a year ago, when a stent was implanted, while a bypass was implanted 12 years ago. Laboratory findings indicate elevated glucose and HbA1c values while other parameters are within reference values. He takes all prescribed medicine alone and on time. I diagnose Capgras syndrome and did psychoeducation. Patient showed an interest in taking medication and a desire for his wife to come back to live with him. Low doses of typical antipsychotic was prescribed, which led to cessation of psychomotor restlessness and harmonization of sleep rhythms. Further neuroradiological diagnostics and regular internist follow-up were recommended.

**Conclusions:**

Previous studies showed the link between Capgras syndrome and aggression, which this case report confirms. Probable basis for emergence of this form of delusional disorder is this patient in not dementia, but rather the consequence of serious organic comorbidities. Further diagnostic processing is in progress.

**Disclosure of Interest:**

None Declared

